# Exploring the Thermal Signature of Guilt, Shame, and Remorse

**DOI:** 10.3389/fpsyg.2020.580071

**Published:** 2020-11-05

**Authors:** Braj Bhushan, Sabnam Basu, Pradipta Kumar Panigrahi, Sourav Dutta

**Affiliations:** ^1^Department of Humanities and Social Sciences, Indian Institute of Technology Kanpur, Kanpur, India; ^2^Department of Mechanical Engineering, Indian Institute of Technology Kanpur, Kanpur, India

**Keywords:** guilt, shame, remorse, thermal change, face

## Abstract

The recent study of complex emotions using visual storyboards by Bhushan et al. (2020) endorses that same scenario can induce guilt/remorse or guilt/shame in people based on valence. These findings were based on behavioral data and did not consider body physiology. The present study aimed to explore the difference in the thermal signature of scenarios that elicit guilt in some and shame/remorse in others. Using storyboard depicting 13 scenarios, we analyzed the thermal changes on the forehead, eyes (left and right separately), cheek (left and right separately), nose tip, and mouth regions of the face with the objective of exploring the thermal signature of guilt, shame, and remorse. Data were collected from 31 participants using a thermal camera in a laboratory setting. We found a difference of 0.5°C or above change in temperature on the forehead, left and right cheeks, and mouth regions during guilt experience compared to shame and remorse experiences. The temperature of the right and left cheeks was high for guilt as compared to remorse for two scenarios inducing guilt/remorse, and the difference was statistically significant. For one of the scenarios inducing guilt/shame, thermal change in the right eye region was higher for shame as compared to guilt. The findings are discussed in light of the distribution of blood vessels on the face.

## Introduction

Although behavioral studies of emotion have largely centered around basic emotions, empirical study of complex emotions, such as guilt, shame, and remorse have also attracted attention of some researchers. Shame has been described as “a highly negative and painful state that also results in the disruption of ongoing behavior, confusion in thought, and inability to speak” (Lewis, 2000, p. 629). According to Tomkins et al. (1995), it represents “the effect of indignity, of defeat, of transgression, and of alienation. [It] is felt as an inner torment, a sickness of the soul” (p. 133).

Emotions soaring out of transgression of cultural ideologies comprise shame/self-condemnation, guilt/remorse, and regret (Fisher and Exline, 2010). Fisher and Exline (2010) have referred to self-condemnation as “an offence-specific version of shame” and remorse as “an offence-specific version of guilt.” The recent study by Bhushan et al. (2020) empirically examined the similarity/dissimilarity among guilt, shame, and remorse. They found that the same scenario induced guilt, shame, or remorse depending on the valence; scenarios with positive factor load resulted into guilt response, whereas those with negative factor load gave rise to shame or remorse. The sign of factor load implies the way the items relate to the factors. Hence, statistically, guilt and shame/remorse were established as opposite poles. They have further argued in the light of indigenous conceptualization of *Lajja* (shame), *aparaadhbodh* (guilt), and *pashchaataap* (remorse), highlighting that such studies are scant. Further, the Western and the Indian connotations of shame (*Lajja*) and guilt is very different. “*Lajja*,” the alike of shame in the Indian context, has been recognized as a positive emotion. “To experience *lajja* is to experience sense of graceful submission and virtuous, courteous well-mannered self of the three emotions—shame, happiness, and anger—Hindu Indians view shame more positively than their European–American counterparts” (Sibia and Misra, 2011, p. 295).

For visualizing the difference or overlap among these three emotions, let us succinctly examine the behavioral assessment tools and how they have operationalized the three complex emotions. Some of the available measures of shame, such as the Revised Shame–Guilt Scale (RSGS; Hoblitzelle, 1987), the Test of Self-Conscious Affect (TOSCA; Tangney et al., 1989), the Other As Shamer Scale (OAS; Goss et al., 1994), the Internalized Shame Scale (ISS; Cook, 1996), and the Experience of Shame Scale (EES; Andrews et al., 2002), consider shame as disposition. On the other hand, some others such as the State Shame and Guilt Scale (SSGS; Marschall et al., 1994) and the Experiential Shame Scale (ExpSS; Turner, 1998) are based on the assumption that being ashamed is a state, and thus, they measure the emotion in a given state.

The Guilt and Shame Proneness scale (GASP; Cohen et al., 2011) measures the propensity to experience guilt and shame for a wide range of personal transgressions. It neither assesses the self-behavior and public–private distinctions nor differentiates between emotional and behavioral response to transgressions. The GASP assesses emotional traits (i.e., guilt proneness and shame proneness) rather than emotional states. The Shame Inventory (Rizvi, 2010) measures propensity to experience shame specific to events as well as overall. Thus, it measures the trait as well as the state. Tangney asserts that the verbal–linguistic representation of shame and guilt does not allow distinction between the two even for educated participants. Further, instead of measuring specific emotional experiences, such tools measure general negative affect (Tangney, 1996; Tangney and Fischer, 1995). TOSCA (Tangney et al., 1989; Tangney, 1990) is a scenario-based measure that assesses shame and guilt. TOSCA-3 (Tangney et al., 2000) accepts the self-behavior distinction. Although it is the widely used tool for assessing guilt and shame proneness, it does not differentiate between negative-behavior evaluations and repair action tendency. Negative self-evaluation and withdrawal action tendencies are also not differentiated (Cohen et al., 2011). Further, the emotional and behavioral responses confound limiting the distinction between these complex emotions. Averilla et al. (2002) have argued that “assessment is constrained by the situations presented in the scenarios” (p. 1373) in TOSCA. Methodologically, two concerns arise out of this—are shame–guilt measures robust enough to assess these constructs, and does high correlation between shame and guilt subscales impact discriminant validity? Further, scores on the available tools of shame are difficult to compare due to their inherent asymmetry. Summarizing the issues with the assessment tools, Rizvi (2010) claims that “many existing measures themselves fail to distinguish these constructs adequately” (p. 438).

As summarized above, the behavioral tools rely on the subjective response of the participants to items or scenarios of a tool. The recent study of Bhushan et al. (2020) suggests difficulty in distinguishing shame and guilt on the basis of events necessitating a relook at these three complex emotions adopting some other technique. Incidentally, physiological studies have reported activation of select brain regions for shame and different regions for guilt, along with some overlap (Takahashi et al., 2004). Thus, it is important to examine the nature and relevant findings of the studies involving behavioral assessment of these emotions vis-à-vis the findings of studies adopting physiological assessment techniques.

Let us now look at the physiological basis of the three complex emotions. The somatic marker hypothesis (Bechara and Damasio, 2005) argues that any event and the corresponding emotion that it generates is linked together by the somatic arousal. Of course, somatic arousal would have a neural underpinning. Normal physiological function involves parasympathetic nervous system (PNS), while the sympathetic system (SNS) regulates physiological changes during heightened emotional states. The 10th cranial nerve or vagus nerve thus prepares the body for the fight–flight response. With few exceptions such as laughter and tear secretion, pleasurable emotions have been associated with parasympathetic activation, whereas negative emotions have been associated with sympathetic arousal.

Musculature analysis of facial expressions of the basic emotions is well established. However, activation of the muscles involves blood flow to the activated muscles, and thus, temperature change during the course of facial expression of emotion might throw light on the thermal signature of emotional state. Change in cutaneous temperature takes place only if subcutaneous blood flows for at least 5 s. This results into heat evasion for 15 s. Thus, there is an average delay of 20 s. This phenomenon applies to dorsal fingertips as well as facial skin (Pavlidis et al., 2001). Thermal imaging can record these observable changes. According to Kuraoka and Nakamura (2011), “the fastest observable change that can be recorded with thermal imaging is 10 seconds.”

Infrared thermography measures temperature changes due to change in blood flow in the activated muscles. Studies have found concordance between Facial Action Coding System (FACS) units and thermography patterns (Jarlier et al., 2011). Infrared thermography was used by Mizukami et al. (1987) and Zajonc et al. (1989) for emotion research. Since then, it has been used by some researchers to explore thermal signature of emotions. Khan et al. (2009) used it for classification of discrete emotions, whereas Robinson et al. (2012) used it to study the dimensional aspects of emotion. Others have studied positive–negative emotion, self-reported high-low arousal (Nhan and Chau, 2010), physiological stress (Krzywicki et al., 2009), stress, fear, and pleasure arousal (Merla and Romani, 2007), and so forth.

Studies suggest that electromagnetic radiation recorded from typical sites such as forehead, eyes, cheeks, tip of nose, and mouth is associated with thermal changes in specific emotion. For instance, Robinson et al. (2012) found negative correlation between temperature of forehead and cheeks and positive emotion. Similarly, increase in temperature around the eyes is an indicator of arousal. Researchers have found increase in temperature around eyes in deception (Pavlidis et al., 2002) and forehead when frustrated (Puri et al., 2005). Fear has been characterized by increase in blood flow to the eyes (300 ms) and a decrease in temperature of the cheeks (Levine et al., 2001; Pavlidis et al., 2001).

Nasal area, for instance, has been found significant for arousal. Nakanishi and Imai-Matsumura (2008) found decline in temperature in the nasal area in infants during joy (following laughter). This has been linked to arousal by Pavlidis et al. (2012). Many researchers have explored the thermal signature of stress. Stress in adults result into increase in blood flow to the frontal region (Puri et al., 2005; Merla and Romani, 2007) and the periorbital regions (Pavlidis and Levine, 2002). However, stress and pain results into overall decrease in facial temperature, particularly in the perioral region. On the other hand, separation stress in infants results into decrease in forehead temperature due to lower arousal (Mizukami et al., 1987, 1990).

Overall study of complex emotions such as guilt, shame, and remorse is sparse, and study of such complex emotions using thermography is rare. We found only one study on guilt, which reported a decline in temperature in the nasal area in guilt (Ioannou et al., 2013). The lack of empirical evidence and the promise of thermography as a technique encouraged us to explore this area.

Taking note of the fact that cultural salience of the life events are significant in the induction of guilt, shame, or remorse as well as the findings of Bhushan et al. (2020) endorsing that the scenarios used in their study with positive factor load resulted into guilt response and only those with negative factor load resulted into shame and remorse response, we selected the scenarios used in their study for the present study, too. It merits mention that such overlap has not only been reported in behavioral studies; rather, the physiological mechanism has also been found to have an overlap. Functional magnetic resonance imaging (fMRI) studies have identified pronounced activation in select regions in the right hemisphere for shame and relatively less activation in both the hemispheres for guilt, especially in the right fusiform gyrus (BA 37), the left middle temporal gyrus (BA 21), the right insula (BA 13), and the amygdala (Michl et al., 2014). However, some overlap has also been reported between shame and guilt, especially in the visual cortex, temporal lobe, and the frontal area (Takahashi et al., 2004). Considering the fact that behavioral as well as neurological studies show some overlap between guilt and shame response and the fact that remorse has not been examined in this context, we attempted to explore the thermal signature of these three complex emotions to answer the following research questions:

RQ-1: Does the thermal signature of the five regions of interest (ROIs) (forehead, eyes, nose tip, cheeks, and mouth) differ among the three storyboards depicting a given scenario respectively for the complex emotion generated by that given scenario?

RQ-2: Does the thermal signature of the five ROIs differ between baseline and poststoryboard (response) conditions respectively for the complex emotion generated by the given scenario?

RQ-3: Does the thermal signature of the five ROIs differ between those scenarios that elicit guilt in some and shame/remorse in others?

## Materials and Methods

### Participants

Thirty-one male participants volunteered for the study. The age of the participants ranged between 19 and 24 years (mean, 20.84 years; SD, 1.21). It merits mention that Bhushan et al. (2020) conducted their study on male participants only. As the present study borrowed the stimulus and the behavioral part of the study from them, the present study was restricted to male participants only. Participation was voluntary, and the study protocol was duly approved by the Institution Ethics Committee of the Indian Institute of Technology Kanpur. They were inducted through an advertisement. The demographic characteristics of the participants were alike with all 31 being native Indians belonging to middle and upper-middle socioeconomic background. The mean of education was 15.86 years (SD, 0.86). All of them were pursuing undergraduate technical course and were good at both spoken and written Hindi and English. They had normal vision and were not using lenses. This was the inclusion criteria. It was ensured that the participants did not suffer from any neurological or psychiatric disorder. None of them were on medication or had caffeine, nicotine, etc. on the day of the experiment. These were the exclusion criteria. The experiment was conducted after obtaining written informed consent from the participants.

### Stimulus

Storyboards of guilt, shame, and remorse scenarios (Bhushan et al., 2020) were shown to the participants. These storyboards comprise of 13 different scenarios, each depicted using three illustrations. A summary of these scenarios along with the complex emotion elicited by them is given in Table 1.

**TABLE 1:** Summary of scenarios (storyboards) and emotions elicited by them.

S. No.	Scenarios	Emotion elicited
1	SB 4	Guilt/Remorse
2	SB 8	Guilt/Remorse
3	SB 26	Guilt/Remorse
4	SB 30	Guilt/Remorse
5	SB 33	Guilt/Remorse
6	SB 9	Guilt/Shame
7	SB 23	Guilt/Shame
8	SB 20	Guilt
9	SB 25	Guilt
10	SB 29	Guilt
11	SB 32	Guilt
12	SB 34	Guilt
13	SB 10	Remorse

Of the total 13 scenarios, 5 have been reported to induce guilt/remorse, 5 guilt, 2 guilt/shame, and 1 remorse, in the participants depending on the nature of the load; while positive load reflects guilt, negative scores reflect shame or remorse. After looking at each storyboard, the participants had to state the emotion elicited in them (shame, guilt, or remorse) and rate it on a 5-point Likert scale (1 = not at all, 5 = very high).

### Procedure

**FIGURE 1:**
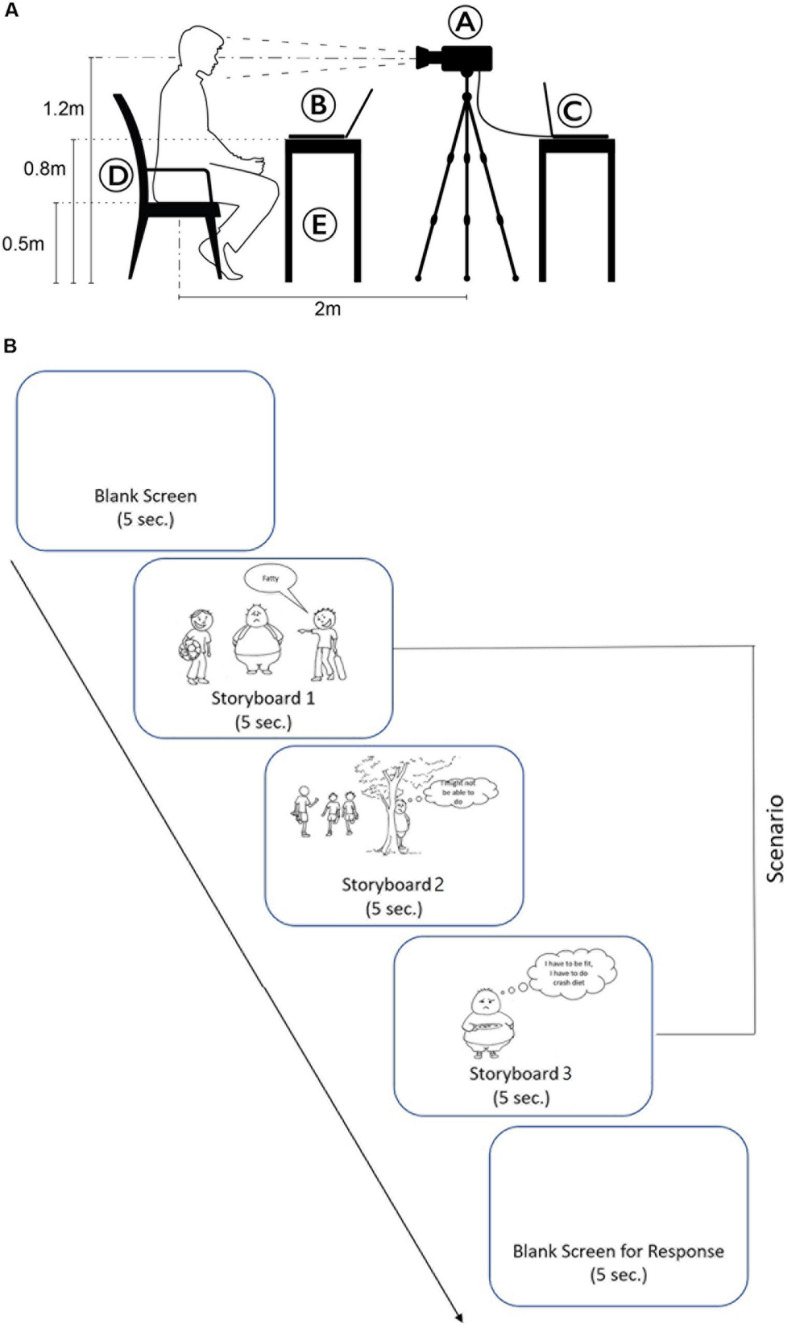
**(A)** Experimental setup (A, thermal camera; B, laptop showing storyboard; C, thermal data recording; D, wooden chair; and E, wooden table). **(B)** Experimental protocol.

Before conducting the experiment, preliminary steps were observed. Our body temperature is affected by the circadian rhythm, and even over a day, it attains the lowest temperature around 4 AM and highest around 4–6 PM. Further, circannual rhythm (change in the season) also affects body temperature. In order to minimize them, we collected data between 4 and 6 PM within a span of 2 weeks during the same season. The temperature of the laboratory was fixed at 26°C. An FLIR SC 5000 thermal camera with the spatial resolution of 640 × 512 pixels, noise equivalent differential temperature (NETD) <20 mK, maximum full-frame rate of 100 frames/s, dynamic resolution of 14 bit, and spectral range of 1.1–5.3 μm was set up at a fixed distance of 2 ft from the chair of the participants. The thermal camera was set to record the face of the participants at the frame acquisition rate of four images per second. A laptop table was kept in front of the chair for presenting the stimulus. The laptop had a 15.6″ (39.62 cm) display and resolution of 1,366 × 768 pixels. The placement of the table was adjusted so that it was properly visible to the participant without obstructing their face from the view of the thermal camera (see Figure 1A). The participants were initially made to sit and relax for 20 min. This time period was required for acclimatization with the laboratory conditions, which is necessary prior to an experiment using a thermal camera. Thereafter, they were briefed about the concepts of guilt, shame, and remorse with the help of illustrated examples (storyboards). After this, the participant was made to sit on a wooden chair facing the thermal camera.

Verbal as well as written instructions were provided to ensure that they understood the concepts of guilt, shame, and remorse, respectively. These three complex emotions were operationally defined based on Proeve and Tudor (2016) as follows:

Guilt (*aparaadhbodh*): Wrongdoing as infringement of a rule or disobeying some imposing command.

Shame (*lajja*): Wrong action as a matter of failing to live up to some close standard that one expected of oneself.

Remorse (*pashchaataap*): Viewing the wrong done as a wrong done to someone, especially where the wrong somehow harms or hurts another person or creature. Figure 1B illustrates the experiment protocol.

According to Pavlidis et al. (2001), cutaneous temperature changes at facial skin takes place only if subcutaneous blood flows for at least 5 s. This results into heat evasion for 15 s with an average delay of 20 s. This guided the stimulus presentation duration in the present experiment. The onset of each storyboard was preceded by a blank screen for 5 s to record the baseline temperature. This was followed by the presentation of the respective storyboards (three illustrations) for 5 s each. Thereafter, the screen turned blank, and the participants had to identify the emotion (guilt, shame, or remorse) induced by the respective storyboard and rate its intensity on a 5-point Likert scale (1 = minimum, 5 = maximum). Participants were instructed beforehand to call out their responses and not move their heads during the course of the experiment. The responses were recorded.

For the purpose of data extraction, the face was divided into five areas of interest (AOIs)—forehead, eyes (left and right separately), cheek (left and right separately), nose tip, and mouth (see Figure 5). The thermal changes on the face were recorded for the full duration of the scenario (blank/baseline until the subjective response). The average temperature during the 5-s window was extracted for all the scenarios where each scenario had five stages (blank, storyboard 1–2–3, and response).

The normal human body temperature ranges between 36.5°C and 37.5°C, and it typically changes by 0.5°C between the highest and lowest points in a day’s time (Mackowiak et al., 1992). As the present study counterbalanced both circadian and circannual rhythms and recorded facial temperatures when the body temperature is likely to be the highest (between 4 and 6 PM), one can anticipate that any further change in the facial temperature would be purely because of the emotion induced by the stimulus.

## Results

**FIGURE 2:**
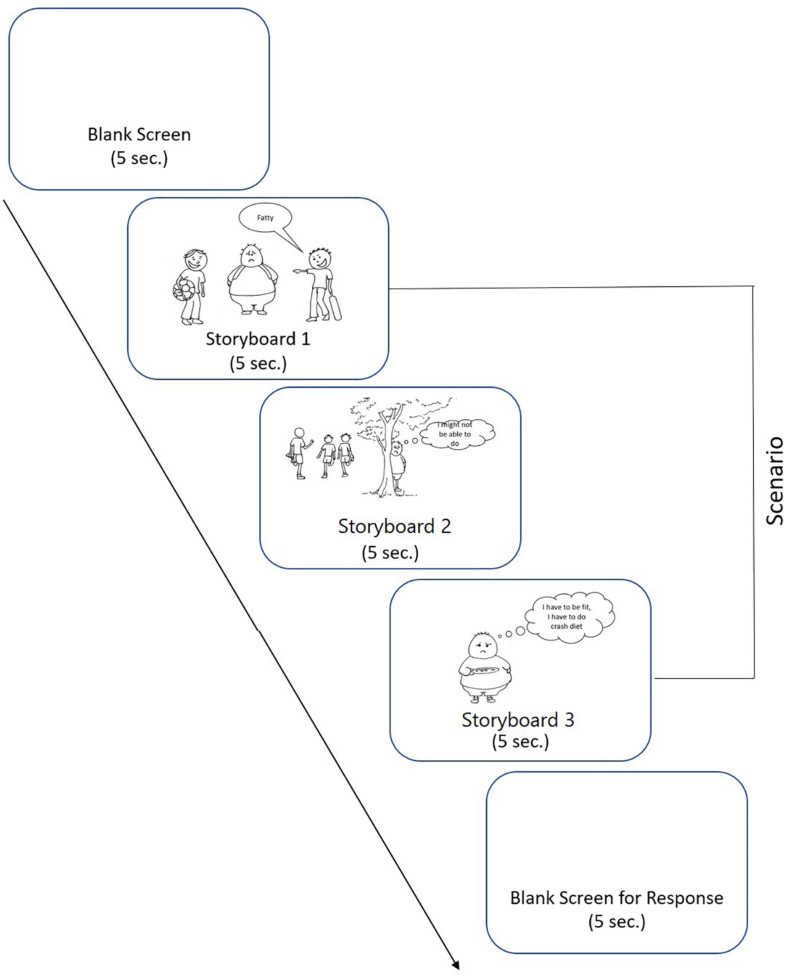
Temperature of the regions of interest (ROIs) across scenarios inducing guilt, shame, and/or remorse.

After extracting corresponding data from all the ROIs, the trend was examined for the three conditions (blank/baseline, storyboard, and response conditions), respectively. Figure 2 illustrates the temperature recorded across ROIs separately for scenarios inducing guilt, remorse, and/or shame. Forehead, nose tip, and mouth seem to be the most happening regions on the face. Temperature change in the eye regions during shame condition shows identical pattern for both the eyes.

The first question at hand (RQ-1) was to find whether the thermal signature of the five ROIs among the three storyboards depicting a given scenario was significantly different or not. To answer this, one-way analysis of variance (ANOVA) was performed comparing the mean temperature of the five AOIs during screening of the three storyboards depicting a scenario. None of the *F*-values were significant, indicating that the thermal signature did not change while the participants were watching the scenarios.

The second question at hand (RQ-2) was to find whether the thermal signature of the five ROIs at the baseline conditions significantly differed from the poststoryboard conditions, respectively. For this, independent sample t test was performed comparing the mean temperature during the pre–postconditions, respectively, for each ROI. None of the t values were significant, suggesting that although the pre–postthermal changes on the face can be seen, they are not significantly higher/lower compared to the previous condition.

The third research question at hand (RQ-3) was to see the difference in the thermal signature of the five ROIs between those scenarios that elicited guilt in some and shame/remorse in others. To answer this question, independent sample t test was performed comparing the mean temperature of each of the five ROIs at the storyboards 1–2–3 and response conditions of those reporting guilt and shame/remorse. Three t values (scenario 4, 33, and 9) were significant (see Table 2).

**TABLE 2:** Significant difference between the regions of interest (ROIs) inducing guilt/remorse or guilt/shame.

Storyboard	ROIs	Emotion	Mean	Sq. Diff.	df	*t*	*p*	Hedges’ *g*
4 (SB-3)	Right cheek	Guilt	34.65	6.03	15,3	2.43	0.05	1.36
		Remorse	33.35	10.25				
33 (SB-1)	Left cheek	Guilt	35.18	3.07	6,12	2.63	0.01	1.23
		Remorse	33.98	13.77				
(SB-2)		Guilt	35.15	2.89	6,12	2.63	0.01	1.23
		Remorse	34.05	11.51				
(SB-3)		Guilt	35.17	3.26	6,13	2.73	0.01	1.26
		Remorse	34.05	11.76				
(Response)		Guilt	35.16	3.05	6,13	2.81	0.01	1.29
		Remorse	34.02	11.42				
9 (SB-3)	Right eye	Guilt	33.73	9.95	24,3	2.92	0.01	1.58
		Shame	34.90	5.03				

Of all the five scenarios inducing guilt/remorse, the cheek region of the face showed significant difference °C°C °C the temperature of the cheek recorded in those reporting guilt and remorse for two scenarios (4 and 33). For scenario 4, the postscenario exposure average temperature of the right cheek (M = 34.64, SD = 0.63) was significantly higher in those who experienced guilt as compared to the average temperature of the right cheek (M = 33.35, SD = 1.85) of those who experienced remorse (*t* = 2.43; df = 15.3; *p* < 0.05; Hedges’ *g* = 1.36).

For scenario 33, the average temperature of the left cheek significantly varied across the baseline to the response conditions. The average temperature of the left cheek during the three-step storyboard as well as the response condition (Table 2) was significantly higher for those experiencing guilt as compared to the average temperature of the left cheek of those experiencing remorse. The robust effect size suggests that the induction of guilt–remorse is likely to affect the temperature of the cheek.

Of the two scenarios inducing guilt/shame, significant difference was found in the temperature of the right eye for scenario 9. The average temperature of the right eye for scenario 9 was significantly higher (M = 34.91, SD = 0.46) in those experiencing shame as compared to those experiencing guilt (M = 32.75, SD = 0.78). This temperature difference was statistically significant (*t* = 2.93; df = 24.3; *p* < 0.006; Hedges’ *g* = 1.59).

**FIGURE 3:**
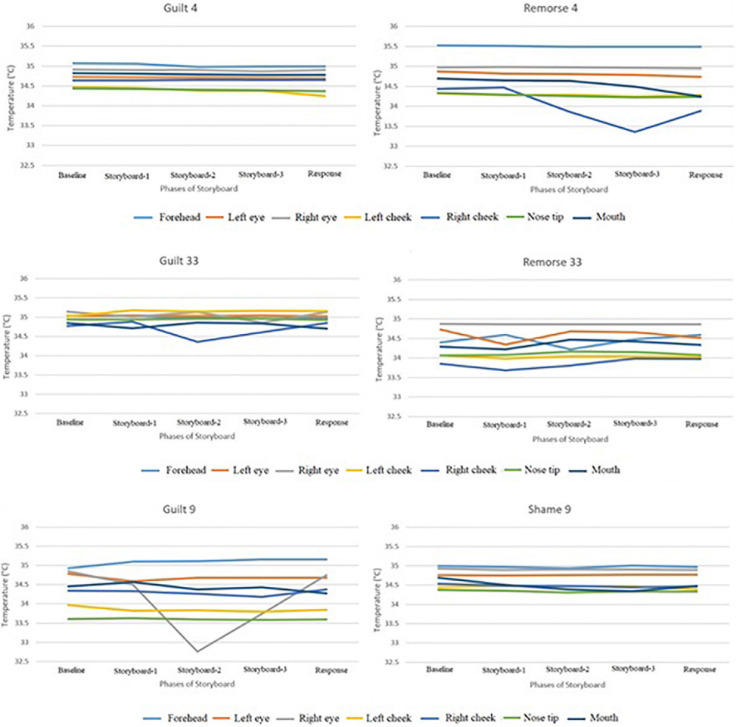
Variation of temperature across the regions of interest (ROIs) for scenarios 4, 33, and 9.

**FIGURE 4:**
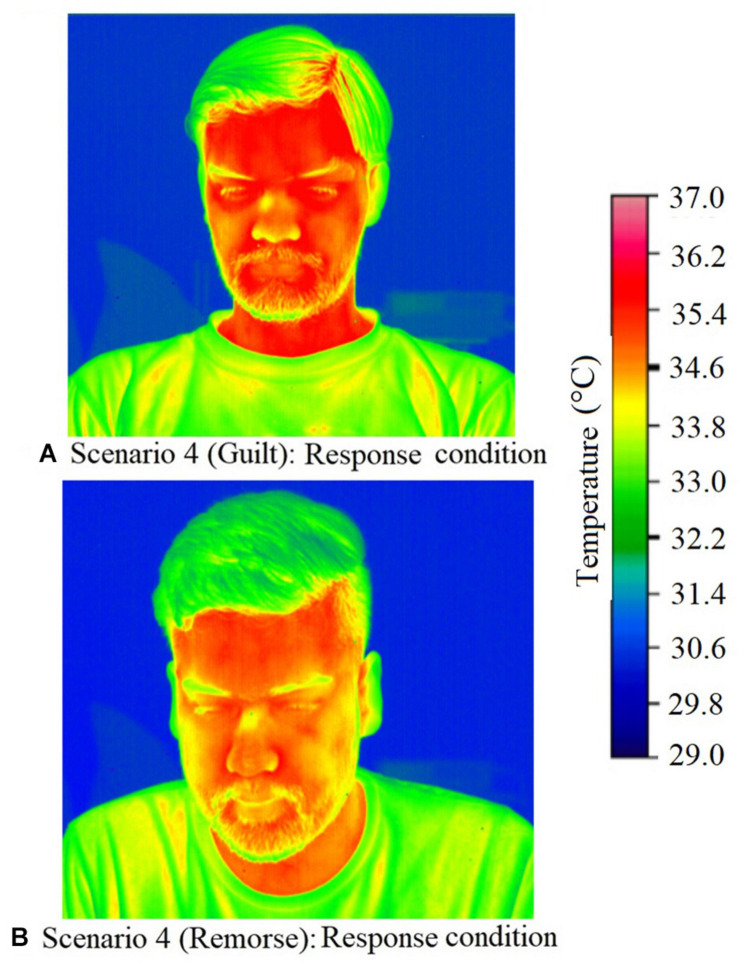
Representative thermal image of **(A,B)** illustrates guilt and remorse conditions on scenario 4 (response condition).

**FIGURE 5:**
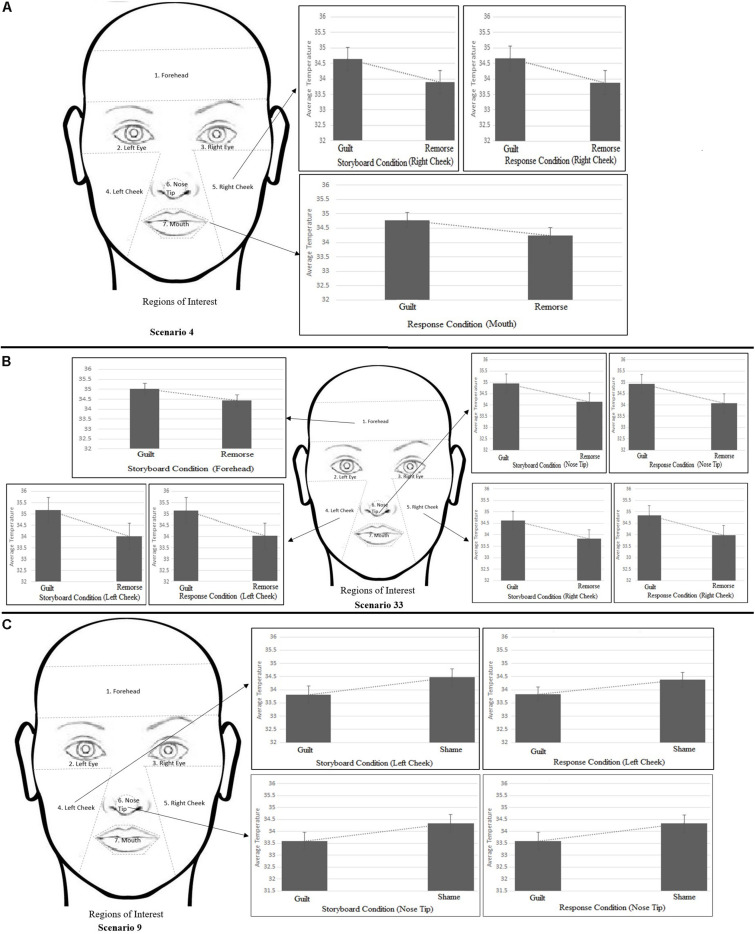
Temperature difference (0.5°C or above) between guilt and shame conditions for guilt/remorse scenarios (4 and 33) and guilt/shame scenario (9). **(A)** shows difference between guilt-shame for scenario 4, **(B)** shows the difference for guilt-shame for scenario 33, and **(C)** shows the difference for guilt-shame for scenario 9.

Figure 3 illustrates the temperature in the respective ROIs for these three scenarios (4, 33, and 9). Figure 4 shows a representative thermal image of two participants, one who experienced guilt and the other who experienced remorse on scenario 4. Figure 5 illustrates the ROI temperature difference (0.5°C or above) between guilt and shame conditions for guilt/remorse scenarios (4 and 33) and guilt/shame scenario (9).

**FIGURE 6:**
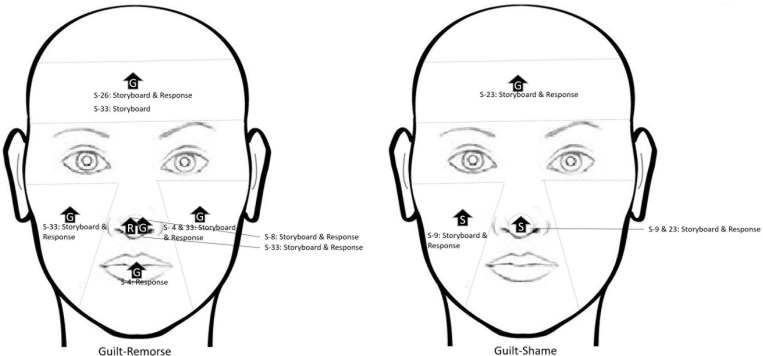
Summary of 0.5°C temperature change across the regions of interest (ROIs) for scenarios inducing guilt/remorse and guilt/shame.

Physiological mechanism of the facial region suggests a difference of 0.5°C change during change in emotional state. Of the 13 scenarios, 5 induced guilt/remorse (scenarios 4, 8, 26, 30, and 33) and 2 induced guilt/shame (scenarios 9 and 23). A 0.5°C temperature change was observed between the storyboard and response conditions in select ROIs for all the scenarios except 30 (see Figure 6). A close look at temperature change beyond 0.5°C reveals higher thermal change during guilt on forehead, cheek (left and right both), and mouth regions compared to shame and remorse. However, the thermal change during guilt on the nose tip was lower than shame. For remorse, the nose tip had mixed results. Comparison of thermal change beyond 0.5°C change for guilt and shame indicates that while guilt increases temperature on the forehead, shame does it on the left cheek. Nose tip did not show any stable pattern; while increase was recorded in one scenario, in the other scenario, it decreased. Guilt induces change in temperature a little slowly on the forehead and mouth; on the right cheek, it brings change a little faster. In majority of the cases, the scenario-induced temperature change lasted until the response phase.

The participants also marked the intensity of the felt emotion on a 5-point Likert scale. The mean of guilt was 13.26 (SD, 5.9), shame was 15.19 (SD, 6.7), and remorse was 13.39 (SD, 6.9). The difference among the three means was statistically not significant.

## Discussion

Some researchers have emphasized the indistinctness between shame and guilt, as several scenarios can engender both (Tangney, 1992), as well as guilt and remorse (Lewis, 1971; Taylor, 1996). The aboriginal idea of *lajja* (shame) is also thought to synthesize both guilt and shame (Bhawuk, 2017). Establishing the empirically drawn fine line of distinction between the three complex emotions has remained a challenge. Based on various statistical analyses, Bhushan et al. (2020) endorsed that same scenario can induce shame/guilt and guilt/remorse in different people. Mapping the similarity/dissimilarity between guilt, shame, and remorse and highlighting the significance of reparation in guilt induction, they argue that “irrespective of the nature of expectations, whether underachieved or unmet, the possibility of amend(s) result into guilt…[and] the absence of scope of restitution results to shame and remorse. Unmet expectations along with lack of reciprocity might lead to the feeling of shame or remorse. On the other hand, the combination of underachieved expectations and lack of reciprocity results only to remorse” (pp. 257–258).

The scenarios used in the present study had factor load ranging between 0.509 and -0.450 (see Bhushan et al., 2020), and the distinction between shame–guilt and guilt–remorse was based on the positive/negative factor load. However, what transpires within during the scenario-induced complex emotion generation cannot be derived from these analyses. The present study fills this gap.

While studying thermal signature of complex emotions, it is imperative that one gives importance to body physiology. Therefore, we extensively analyzed the 0.5°C thermal change in ROIs even if they were statistically not significant. According to Ariyaratnam and Rood (1990), the temperature on various regions of the face range between 34°C (forehead region) and 32°C (cheek region). Our data show a difference of 0.5°C change in temperature on forehead, left and right cheeks, and mouth regions during guilt experience compared to shame and remorse experiences. Guilt experience resulted into higher temperature. For the two scenarios inducing guilt/shame, a 0.5°C thermal change was recorded in the forehead and left cheek; while guilt experience increased temperature on the forehead, shame experience did so on the left cheek. As far as nose tip is concerned, the thermal change during guilt experience was lower than shame. On the other hand, the nose tip had mixed results for remorse experience; while an increase was recorded in one scenario, in the other scenario, it decreased. The nasal area has been reported to exhibit arousal (Nakanishi and Imai-Matsumura, 2008). As stated earlier, we found only one thermography-based study of guilt (Ioannou et al., 2013) that reported a decline in temperature in the nasal area in guilt, whereas we got inconsistent results with respect to the nose tip. The thermal signature of complex emotion on the face is dependent on the branching of blood vessels on the face, orbit (eye socket), and the nasal cavity. Facial artery is a branch of the external carotid artery, and it is the main source of blood supply to the face. Some other smaller arteries such as submandibular, inferior and superior labial, angular, supraorbital, superficial temporal, transverse facial, and supratrochlear arteries also carry blood to the face. Thus, change in facial temperature is a by-product of variation in blood flow, which in turn is affected by the arousal level triggered by the scenarios inducing shame, guilt, or remorse. This study unambiguously endorses this.

For two of the five scenarios inducing guilt/remorse, the thermal change in the cheek region of the face was statistically significant. Having been exposed to the storyboard of scenario 4, the temperature of the right cheek of those who experienced guilt was significantly higher compared to those who experienced remorse. For scenario 33, average temperature of the left cheek during the three-step storyboard as well as the response condition was significantly higher for those experiencing guilt as compared to those experiencing remorse. The thermal signature of guilt–remorse experience seems to affect the cheek region. Of the two scenarios inducing guilt/shame, thermal change induced by scenario 9 resulted into a significant difference in the right eye region wherein the temperature was higher in those experiencing shame as compared to those experiencing guilt. The available literature suggests rise in temperature in the eye region in deception (Pavlidis et al., 2002) and in the forehead in frustration (Puri et al., 2005). Due to dearth of empirical evidence pertaining to the thermal signature of complex emotions, the findings of this study highlight the promise of thermography as a technique to study complex emotions.

The subjective rating of shame was relatively higher compared to guilt and remorse. However, this difference was statistically not significant. Zhu et al. (2015) have reported difference in the time course of processing facial expressions and intensity assessment. However, we did not come across any study on time course analysis for complex emotions.

The findings of this study are significant to distinguish the three complex emotions, viz. guilt, shame, and remorse. The mix of behavioral method and thermography techniques is still not so commonly reported in emotion research. Although small sample size limits the generalization of the findings, it does throw light on the otherwise overlapping complex emotions. The findings of this study extend unequivocal support to the significance of thermal signature of complex emotions, as it could counterbalance both circadian and circannual rhythms. However, this technique has a limitation. Despite its technological sophistication, thermography has certain limitations when used for emotion research. Although it has been largely used to study basic emotions and researchers have identified specific facial area showing thermal change with respect to specific emotions, it has limitations in distinguishing emotions involving the same muscle group.

## Data Availability Statement

The raw data supporting the conclusions of this article will be made available by the authors, without undue reservation.

## Ethics Statement

The studies involving human participants were reviewed and approved by the Institution Ethics Committee, Indian Institute of Technology Kanpur. The patients/participants provided their written informed consent to participate in this study.

## Author Contributions

All authors equally contributed to the planning and conduct of the study. SB and SD collected the data, while BB and PP supervised the data acquisition process. BB analyzed the data and drafted the first version of the manuscript, and all other authors gave their inputs while finalizing it. All authors agreed to be accountable for the content of the work.

## Conflict of Interest

The authors declare that the research was conducted in the absence of any commercial or financial relationships that could be construed as a potential conflict of interest.
